# Localized surface-based morphometric differences in painful temporomandibular disorder

**DOI:** 10.1016/j.ynirp.2026.100404

**Published:** 2026-09-10

**Authors:** Soamy Montesino-Goicolea, Pedro Antonio Valdes-Hernandez, Julio A. Peraza, Margarete Ribeiro Dasilva, Stephen A. Coombes, Yenisel Cruz-Almeida

**Affiliations:** a Pain Research & Intervention Center of Excellence, University of Florida, Gainesville, FL, USA; b Department of Community Dentistry & Behavioral Science, College of Dentistry, University of Florida, Gainesville, FL, USA; c Center for Cognitive Aging & Memory, McKnight Brain Foundation, University of Florida, Gainesville, FL, USA; d Division of Clinical and Population Health Integration, Department of Health Outcomes and Biomedical Informatics (HOBI), College of Medicine, University of Florida, Gainesville, FL, USA; e Department of Physics, Florida International University, Miami, FL, USA; f Department of Restorative Dental Science, Prosthodontics Division, College of Dentistry, University of Florida, Gainesville, FL, USA; g Department of Applied Physiology and Kinesiology, University of Florida, Gainesville, FL, USA

**Keywords:** Temporomandibular disorder, Surface-based morphometry, Anterior cingulate cortex, Cortical curvature, Chronic pain, FreeSurfer, Neuroimaging

## Abstract

Temporomandibular disorders (TMDs) are prevalent chronic orofacial pain conditions associated with functional impairment and reduced quality of life. This cross-sectional brief report examined cortical and subcortical morphometric differences between 19 individuals with painful TMD (p-TMD) and 20 age- and sex-matched pain-free controls using high-resolution T1-weighted MRI and FreeSurfer-based surface morphometry. Compared with controls, p-TMD participants showed greater cortical curvature and volume within the left anterior and mid-anterior parts of the cingulate gyrus and sulcus, respectively. No additional cortical or subcortical differences survived correction for multiple comparisons. These findings provide preliminary evidence of localized cingulate cortex morphometric differences in p-TMD, consistent with this region's role in pain-related attentional and sensorimotor processes.

## 1 Introduction

Temporomandibular disorders (TMDs) are among the most prevalent chronic musculoskeletal pain conditions, affecting approximately 5–10% of the general population, with a higher prevalence among women. These disorders are characterized by persistent orofacial pain and functional limitations, such as restricted jaw movement, that substantially impair quality of life and impose significant socioeconomic costs worldwide (National Academies of Sciences E and MH and MDB et al., 2020; Zieliński et al., 2024). Despite their clinical impact, the neurobiological mechanisms underlying painful TMD (p-TMD) remain incompletely understood.

Historically, TMD has been conceptualized primarily as a peripheral disorder involving the temporomandibular joint and masticatory musculature. However, accumulating evidence indicates that central nervous system processes contribute to the persistence and modulation of pain in TMD (Sarlani and Greenspan, 2005; Zhao et al., 2025). Structural neuroimaging studies across chronic pain conditions—including fibromyalgia, chronic low back pain, and migraine—have identified alterations in brain regions involved in nociceptive processing, affective regulation, and motor control, including the prefrontal cortex, insula, and cingulate cortex (Apkarian et al., 2004; DaSilva et al., 2007; Rodriguez-et al., 2009; Smallwood et al., 2013). In TMD specifically, neuroimaging findings have been heterogeneous. While some voxel-based morphometry (VBM) studies have reported gray matter reductions in pain-related regions, others have found no significant group differences (Domin et al., 2021). More recent surface-based morphometry (SBM) investigations have similarly yielded mixed results, including reports of preserved cortical thickness or localized increases in frontal and cingulate regions (Li et al., 2025a, 2025b).

Compared with volumetric approaches, SBM offers a more detailed characterization of cortical architecture by independently quantifying morphometric features such as cortical thickness, surface area, curvature, and volume—metrics that are often conflated in global gray matter volume analyses (Li et al., 2025b). Among these, cortical curvature quantifies the folding geometry of the cortical surface and has been shown to be sensitive to cytoarchitectural reorganization in neurological conditions (Deppe et al., 2014; King et al., 2016), yet it has rarely been examined in TMD. Examination of these complementary features may help reconcile inconsistencies in the literature and clarify whether chronic pain conditions are associated with uniform cortical atrophy or with localized, feature-specific structural differences.

Despite these methodological advances, important gaps remain. Relatively few studies have applied contemporary surface-based pipelines to examine multiple cortical morphometric features in carefully age- and sex-matched TMD samples, and even fewer have focused on curvature- and volume-based metrics within pain-related cortical networks. Moreover, it remains unclear whether structural differences in TMD extend to subcortical structures such as the thalamus, hippocampus, and amygdala—regions in which volumetric alterations have been associated with pain duration in TMD (Domin et al., 2021) and reported in fibromyalgia and chronic low back pain (Apkarian et al., 2004; Rodriguez-et al., 2009).

In this brief report, we used high-resolution structural MRI to examine both cortical and subcortical morphometric differences between individuals with painful TMD and age- and sex-matched pain-free controls. Cortical analyses were spatially constrained to a meta-analytic pain-related mask on theoretical grounds, but were otherwise exploratory: no specific region, morphometric feature, or direction of effect was prespecified, and all seven surface-based measures were examined on equal footing. Subcortical analyses compared volumes of structures implicated in the chronic pain literature. Our objective was to characterize the distribution of morphometric differences across the pain network in p-TMD and to generate regionally specific hypotheses for testing in larger, adequately powered samples.

## 2 Methods

The study was approved by the University of Florida Institutional Review Boards (IRB #411-2007). All participants provided written informed consent prior to participation, and all procedures were conducted in accordance with the Declaration of Helsinki.

### 2.1 Participants

The present work is a cross-sectional secondary analysis of structural MRI data acquired during a parent study of chronic jaw pain and sensorimotor function conducted at the University of Florida, from which behavioral, fMRI, and EEG findings have been reported previously (Wang et al., 2018, 2019, 2020). Recruitment and data collection occurred between February 4, 2009, and June 28, 2011. A total of 39 individuals were included in the present analyses: 19 participants with chronic painful temporomandibular disorder (p-TMD) and 20 age- and sex-matched pain-free healthy controls. Sample size was determined by the number of parent-cohort participants with usable structural MRI rather than by an a priori power calculation; for context, a two-tailed independent-samples comparison at this sample size (α = .05) provides 80% power to detect an effect of Cohen's d = 0.92, indicating sensitivity to large effects only. All analyses are therefore exploratory rather than confirmatory, and the findings require replication in adequately powered samples.

Participants were recruited under a single institutional protocol that supported parallel electroencephalography and magnetic resonance imaging studies, and not all enrolled individuals took part in the imaging session. The present analyses include every participant from that protocol for whom a usable structural T1-weighted scan was available; no participant was excluded after image acquisition.

Participants in the p-TMD group were identified using a standardized TMD pain-screening questionnaire consistent with the Research Diagnostic Criteria for TMD (RDC/TMD) (Gonzalez et al., 2011; Schiffman et al., 2014). Participants were required to meet all of the following criteria: (i) persistent unilateral or bilateral pain in the jaw/temporal region during the preceding 30 days; (ii) self-reported jaw pain or stiffness upon awakening; and (iii) endorsement of pain modulation during at least three categories of routine activities. A clinical examination in accordance with RDC/TMD Axis I was not performed, and diagnostic subtypes were therefore not determined. Control participants were excluded if they reported a history of neurological or psychiatric disorders, chronic pain conditions, or any ongoing acute pain at the time of assessment. Symptom duration was not applied as a separate eligibility threshold; among the 16 p-TMD participants for whom duration was recorded, jaw pain had been present for 14.13 ± 8.87 years (range 3–30), consistent with the chronic samples described in previous reports from this cohort (Wang et al., 2018, 2019, 2020).

Comorbid chronic pain conditions were recorded at intake by self-report. Twelve of the 19 p-TMD participants reported none. Of the remaining seven, four reported a single condition and three reported two, giving ten reported conditions in total: migraine (five participants), fibromyalgia (two), rheumatoid arthritis (one), osteoarthritis (one) and a connective-tissue disorder (one). Such conditions were not exclusionary for the p-TMD group, consistent with the eligibility criteria applied in the parent study (Wang et al., 2018, 2019, 2020), in which exclusion criteria were specified for the control group only. The potential contribution of comorbid chronic pain to the morphometric findings is addressed in the Limitations. Demographic and clinical characteristics are summarized in Table 1.

**Table 1:** Group demographics, chronic pain measures and psychological measures for the chronic jaw pain group and the pain-free healthy controls.

	Control n = 20	TMD n = 19	Test Statistic (p)
Age	30.80 ± 11.98	36.47 ± 14.99	t(34.4) = 1.30, p = .202
Sex, No (%)			χ^2^(1) = 0.74, p = .389
Female	9 (45%)	6 (32%)	
Male	11 (55%)	13 (68%)	
Race, No (%)			χ^2^(5) = 5.45, p = .363
Undefined	1 (5%)	0 (0%)	
African American	0 (0%)	2 (11%)	
Asian	2 (10%)	1 (5%)	
Caucasian	15 (75%)	13 (68%)	
Hispanic	1 (5%)	3 (16%)	
Pacific Islander	1 (5%)	0 (0%)	
Duration of Pain (years) n = 16 (3 missing values)	0	14.13 ± 8.87	
Chronic pain measures			
GCPS characteristic pain intensity (0–100)	1.33 ± 3.32	42.72 ± 14.90	t(19.7) = 11.83, p < .001
GCPS pain interference (0–100)	0.00 ± 0.00	16.91 ± 22.15	t(18.0) = 3.33, p = .004
GCPS disability score (0–3)	0.00 ± 0.00	0.37 ± 0.83	t(18.0) = 1.93, p = .069
JFLS-20 mastication sum (0–60)	0.40 ± 1.05	13.26 ± 8.07	t(18.6) = 6.89, p < .001
JFLS-20 jaw mobility sum (0–40)	0.20 ± 0.89	8.74 ± 7.33	t(18.5) = 5.04, p < .001
OBC total (0–84)	15.73 ± 8.84	30.94 ± 7.36	t(36.4) = 5.85, p < .001
TSK-17 total (17–68)	30.19 ± 6.24 (n = 18)	34.63 ± 4.75	t(31.8) = 2.43, p = .021
Psychological measures (PROMIS raw sums)			
Fatigue (0–32)	6.65 ± 5.49	13.21 ± 8.22	t(31.2) = 2.92, p = .007
Anxiety (0–32)	4.00 ± 3.32	8.79 ± 6.20	t(27.3) = 2.99, p = .006
Depression (0–32)	2.00 ± 3.36	3.58 ± 4.21	t(34.4) = 1.29, p = .205

Note. Values are mean ± SD unless otherwise indicated; group comparisons are two-tailed Welch's t-tests and categorical comparisons chi-square tests. PROMIS scores are raw item sums, and missing-data handling is described in Section 2.2. Control participants were pain-free by eligibility criteria; the small nonzero values on pain-related instruments reflect minimal symptoms reported by three participants at assessment, and no control reported pain interference or disability. p-TMD = painful temporomandibular disorder; GCPS = Graded Chronic Pain Scale; JFLS-20 = Jaw Functional Limitation Scale, 20-item; OBC = Oral Behaviors Checklist; TSK-17 = Tampa Scale for Kinesiophobia, 17-item; PROMIS = Patient-Reported Outcomes Measurement Information System.

### 2.2 Clinical and behavioral measures

Self-reported pain duration (in years) was recorded for each p-TMD participant. Pain severity was assessed using the Graded Chronic Pain Scale (GCPS) (Von et al., 1992). Jaw-related functional limitation was evaluated using the Jaw Functional Limitation Scale (JFLS) (Ohrbach et al., 2008). Oral parafunctional behaviors were quantified using the Oral Behaviors Checklist (OBC) (Markiewicz et al., 2006). Pain-related fear of movement was assessed with the Tampa Scale for Kinesiophobia (TSK) (Miller et al., 1991). Psychological symptoms were evaluated using the Patient-Reported Outcomes Measurement Information System (PROMIS) short forms for fatigue, anxiety, and depression.

Scale details: The GCPS yields characteristic pain intensity score and a pain interference score (each 0–100, computed as the mean of three 0–10 ratings multiplied by 10; higher scores indicate greater severity) and a disability score (0–3; higher scores indicate greater disability-related impairment). The JFLS 20-item version was used; the mastication subscale (items 1–6) and mobility subscale (items 7–10) were analyzed separately as item sums (possible ranges 0–60 and 0–40 respectively; higher scores indicate greater functional limitation; internal consistency α > .90 in orofacial pain populations). The OBC total score was computed from 21 items rated on a 0–4 frequency scale (higher scores indicate more frequent parafunctional behaviors; α ≈ .82). The TSK total score ranges from 17 to 68 (17 items rated 1–4; higher scores indicate greater pain-related fear of movement; α ≈ .77). PROMIS 8-item short forms were analyzed as raw summed scores (8 items rated 0–4; possible range 0–32; higher scores indicate greater symptom burden α > .90 for each domain). Missing or out-of-range item responses were handled uniformly across instruments: where at least 80% of items were usable, the total was prorated to the full scale; where fewer were usable, the total was treated as missing. Two control participants completed 8 and 4 of the 17 TSK items and were therefore excluded from that comparison, and a third completed 16 of 17 and was prorated; three control participants and one p-TMD participant had a single unusable OBC item and were prorated likewise. Sample sizes for each measure are reported in Table 1.

### 2.3 MRI data acquisition and preprocessing

MRI data were acquired at the McKnight Brain Institute (University of Florida) using a 3-T Philips Achieva system equipped with a 32-channel phased-array head coil. High-resolution structural images were obtained using a T1-weighted MP-RAGE sequence (TR/TE/TI = 7/3.2/2750 ms; flip angle = 8°; FOV = 240 × 240 mm; 1 mm isotropic voxels across 170 sagittal slices). Structural images were processed using FreeSurfer (version 7.3.2; recon-all). Seven vertex-wise surface-based morphometric measures were derived: cortical thickness, cortical surface area, cortical volume, pial surface area, sulcal depth, smoothed mean curvature, and white-matter Jacobian determinant.

Image quality was assessed for all 39 datasets. No image-quality exclusions were applied a priori, and no scan required manual correction: no pial or white-matter boundary edits and no control points were applied to any reconstruction. Reconstruction quality was instead quantified retrospectively using the Euler number, a summary index of surface-reconstruction quality that tracks expert manual ratings (Rosen et al., 2018), together with Talairach registration quality and area-weighted gray/white contrast and its signal-to-noise ratio; groups were compared on each metric with false discovery rate correction and adjustment for age.

### 2.4 Surface-based cortical morphometric analysis

Between-group differences in cortical morphometry were examined using general linear models (GLMs) implemented in FreeSurfer's mri_glmfit. Analyses were constrained to a meta-analytic Neurosynth “pain” mask (Yarkoni et al., 2011), binarized at z > 1.96 and transformed to fsaverage surface space using mri_surf2surf. Cluster-level inference used permutation-based correction (5000 permutations; vertex-wise threshold p < .001; cluster-wise p < .05, Bonferroni-adjusted across both hemispheres). Surface maps were smoothed with a 10 mm full-width at half-maximum Gaussian kernel prior to analysis. Age was entered as a covariate in all models, and estimated total intracranial volume (eTIV) additionally for the volume measure. Anatomical localization was performed using the Destrieux parcellation (aparc.a2009s) (Destrieux et al., 2010).

Several features of the design limit potential sources of bias. Groups were matched on age and sex at recruitment; all images were acquired on a single scanner with an identical head coil and acquisition protocol throughout the recruitment period; and all scans were processed through an identical automated pipeline, with cluster-level inference corrected by permutation across both hemispheres. Because case status was determined before imaging and all morphometric processing was automated, ascertainment of the outcome was independent of group assignment.

### 2.5 Subcortical volume analysis

Volumes of seven bilateral subcortical structures (14 total: thalamus, hippocampus, amygdala, caudate nucleus, putamen, globus pallidus, and nucleus accumbens) were compared between groups using separate ANCOVAs, with age and eTIV entered as covariates. Multiple comparisons were controlled using the Benjamini–Hochberg false discovery rate (FDR) procedure (Benjamini and Hochberg, 1995), with a significance threshold of q < 0.05. Effect sizes are reported as Hedges’ g with 95% confidence intervals.

### 2.6 Exploratory clinical correlations

Within the p-TMD group, exploratory Pearson correlations were computed between the cluster-average morphometric values extracted from each significant cortical cluster and the following clinical measures: pain duration, GCPS pain intensity, GCPS pain interference, disability score, JFLS mastication subscale, JFLS mobility subscale, OBC total score, TSK total score, and PROMIS fatigue, anxiety, and depression scores. All correlations were evaluated at an uncorrected threshold of p < .05, given the exploratory nature of this analysis. Subcortical ANCOVAs and Pearson correlations were performed using IBM SPSS Statistics (IBM Corp., Armonk, NY, USA).

## 3 Results

### 3.1 Sample characteristics

Demographic and clinical characteristics are presented in Table 1. Mean age was 36.47 (SD = 14.99) years in the p-TMD group and 30.80 (SD = 11.98) years in the control group; groups did not differ significantly on age (t(34.4) = 1.30, p = .202). The p-TMD group comprised 6 women and 13 men; the control group comprised 9 women and 11 men; sex distribution did not differ between groups (χ^2^(1) = 0.74, p = .389, V = 0.14). Racial composition was also comparable across groups (χ^2^(5) = 5.45, p = .363). Among p-TMD participants with available data, mean pain duration was 14.13 ± 8.87 years (range: 3–30 years; n = 16; three participants had missing pain duration values).

### 3.2 Surface-based cortical morphometric differences

**Fig. 1:**
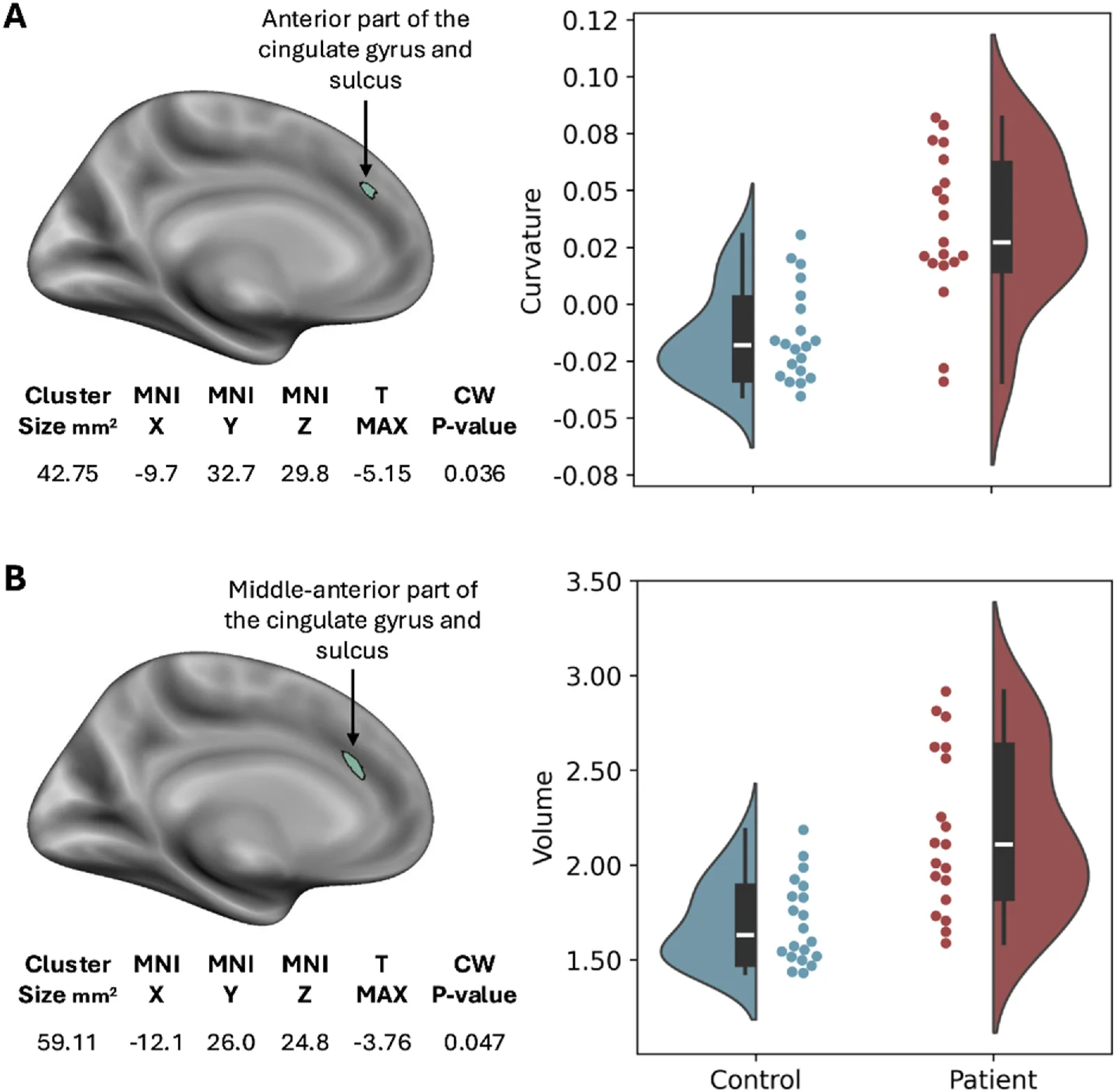
Surface-based cortical morphometric differences in painful TMD. Vertex-wise surface-based analyses revealed localized differences in (A) the cortical curvature of the anterior part of the cingulate gyrus and sulcus and in (B) the cortical volume of the left mid-anterior cingulate gyrus and sulcus, in individuals with painful temporomandibular disorder (p-TMD) compared with pain-free controls. These regions were defined by the Destrieux sulcal-gyral parcellation (FreeSurfer aparc. a2009s (Destrieux et al., 2010)). Significant clusters are displayed on the FreeSurfer fsaverage cortical surface. For each cluster, surface area, MNI coordinates of the vertex exhibiting the peak t-statistic, maximum t-value, and cluster-wise corrected p-value are reported. Violin plots illustrate the distribution of morphometric values at the vertex corresponding to the peak statistical effect, with individual data points overlaid. p-TMD painful temporomandibular disorder.

Between-group analyses revealed significantly greater cortical curvature in p-TMD compared with controls, localized to a cluster encompassing the left anterior cingulate gyrus and sulcus (peak Destrieux label: G_and_S_cingul-Ant; max |t| = 5.15; cluster surface area = 42.75 mm^2^; peak MNI: −9.7, 32.7, 29.8; cluster-wise corrected p = .036; Table 2, Fig. 1A). At the peak vertex, the p-TMD group exhibited higher curvature values (M = 0.013, SD = 0.031) than controls (M = −0.026, SD = 0.019). Greater cortical volume in p-TMD was similarly identified within a cluster encompassing the left mid-anterior cingulate gyrus and sulcus (peak Destrieux label: G_and_S_cingul-Mid-Ant; max |t| = 3.76; cluster surface area = 59.11 mm^2^; peak MNI: −12.1, 26.0, 24.8; cluster-wise corrected p = .047; Table 2, Fig. 1B). At the peak vertex, p-TMD participants showed higher cortical volume values (M = 1.797, SD = 0.499) relative to controls (M = 1.446, SD = 0.273). No significant between-group differences were identified for the remaining five morphometric measures: cortical thickness, surface area, pial surface area, sulcal depth, or white-matter Jacobian determinants.

**Table 2:** Significant cortical morphometric clusters: p-TMD vs. controls.

Measure	Hemi	Destrieux Label	Size (mm^2^)	MNI [x, y, z]	Peak t (in absolute value	N Vert.	CWP
Curvature	Left	G_and_S_cingul-Ant	42.75	−9.7, 32.7, 29.8	-5.15	64	0.036
Volume	Left	G_and_S_cingul-Mid-Ant	59.11	−12.1, 26.0, 24.8	-3.76	112	0.047

Note. Hemi = hemisphere; MNI = Montreal Neurological Institute peak coordinates; CWP = cluster-wise corrected p-value (permutation-based, Bonferroni-adjusted across both hemispheres); p-TMD > controls for both measures. Destrieux labels from FreeSurfer aparc.a2009s.

### 3.3 MRI quality diagnostics

All reconstructions completed without error, and the Euler number had a median of −14 (range −186 to −6), with no participant falling below the exclusion threshold of −217 established by Rosen et al. (2018). The p-TMD and control groups did not differ on any quality metric (all |Hedges' g| ≤ 0.21, all p_FDR_ ≥ 0.99; all p ≥ .63 after adjustment for age, as in the group model). A sample-relative criterion (Euler number below the lower Tukey fence) would have flagged three datasets, two p-TMD and one control; all were retained, as no dataset met the absolute threshold and quality did not differ between groups. Image quality is therefore unlikely to account for the reported group effects (Rosen et al., 2018).

### 3.4 Subcortical volumes

No significant between-group differences in subcortical volumes survived FDR correction (all q > 0.68). Effect sizes were uniformly small across all seven bilateral structures (|Hedges’ g| < 0.35). The largest uncorrected group difference was observed for the right thalamus (g = 0.345; uncorrected p = .069, q = 0.685). Effect sizes for the remaining structures—hippocampus, amygdala, caudate, putamen, globus pallidus, and nucleus accumbens—were all |g| ≤ 0.30.

### 3.5 Exploratory clinical correlations

Exploratory Pearson correlations within the p-TMD group revealed no significant associations between cluster-average morphometric values and any of the clinical measures examined (all ps > 0.05). For the curvature cluster, correlations ranged from r = −0.304 (depression; p = .205) to r = 0.225 (JFLS mobility; p = .356). For the volume cluster, correlations ranged from r = −0.162 (disability score; p = .507) to r = 0.246 (JFLS mobility; p = .311). Associations between cingulate cluster values and pain duration were also non-significant (curvature cluster: r = 0.164, p = .544; volume cluster: r = −0.003, p = .993).

## 4 Discussion

The present study identified localized cortical morphometric differences in individuals with painful temporomandibular disorder compared with age- and sex-matched pain-free controls. Specifically, greater cortical curvature and volume were observed within the left anterior and mid-anterior parts of the cingulate gyrus and sulcus, respectively, in the absence of widespread cortical or subcortical structural alterations. These findings are consistent with the emerging view that chronic orofacial pain is associated with regionally specific cortical reorganization rather than diffuse gray matter reductions.

The observed pattern of greater cortical metrics in the cingulate—rather than reductions—diverges from earlier VBM reports that emphasized gray matter loss in pain-related regions (Apkarian et al., 2004; Rodriguez-et al., 2009) but aligns with more recent SBM investigations showing preserved or increased cortical thickness and volume in frontal and cingulate regions in TMD (Li et al., 2025a, 2025b). This divergence may reflect methodological differences between VBM and SBM approaches, the specific morphometric features examined, and sample characteristics including TMD subtype and chronicity. Importantly, cortical curvature has been proposed as a sensitive index of cytoarchitectural organization in neurological conditions (Deppe et al., 2014; King et al., 2016), although the processes giving rise to curvature differences cannot be established from cross-sectional structural imaging. A recent individual participant data meta-analysis (N = 401 across five chronic pain conditions) reported significant extrinsic curvature increases in the right caudal anterior cingulate cortex (Loke et al., 2025), lending convergent validity to the present finding and suggesting that cingulate curvature changes may represent a transdiagnostic neurobiological signature of chronic pain. Notably, the present finding was lateralized to the left hemisphere, whereas the meta-analytic effect reported by Loke et al. (2025) was observed in the right caudal ACC. This hemispheric discrepancy may reflect differences in ACC subdivision boundaries, sample composition, or the specific morphometric features examined, and warrants investigation in larger, adequately powered studies.

The mid-anterior cingulate cortex (aMCC) plays a central role in pain-related attentional control, sensorimotor integration, and action selection (Misra and Coombes, 2015; Shackman et al., 2011; Strohman and Legon, 2025). Structural differences within this region are therefore theoretically consistent with documented difficulties in the attentional and volitional aspects of pain modulation reported in individuals with TMD and related chronic orofacial pain conditions (Wang et al., 2018, 2019, 2020). Greater cortical volume and curvature in the aMCC may reflect structural remodeling associated with ongoing nociceptive input, although whether such differences are adaptive, maladaptive, or incidental cannot be determined from the present data. The cellular substrates of macrostructural differences of this kind cannot be identified from T1-weighted imaging. Candidate mechanisms proposed in the animal and human literature, including dendritic remodeling, glial changes, and altered cortical folding under sustained afferent drive (Metz et al., 2009; Pomares et al., 2017), remain speculative and would require histological or multimodal quantitative imaging to test.

The absence of significant subcortical volumetric differences after FDR correction—despite the right thalamus showing the largest uncorrected group difference (Hedges’ g = 0.345)—may reflect limited statistical power given the modest sample size or may indicate that subcortical involvement in TMD is more functionally than structurally manifest at this stage of the condition. It is also possible that subcortical structural changes emerge only at later or more severe stages of pain chronification; considerable variability in pain duration (3–30 years) within the present p-TMD sample may have obscured such effects.

The absence of significant within-group brain–behavior correlations between cingulate morphometric values and clinical measures is consistent with prior small-sample neuroimaging studies of chronic pain. Reproducible brain–behavior associations in this domain typically require samples of several hundred to several thousand participants (Marek et al., 2022). Given the present sample size of 19 p-TMD participants, the current correlational results should be interpreted as preliminary and hypothesis-generating rather than conclusive.

The finding of structural differences within the aMCC has translational relevance. The aMCC is critically involved in the attentional and motivational aspects of pain processing, action selection under aversive conditions, and sensorimotor integration. Structural differences in this region provide a neurobiological rationale for rehabilitation approaches that explicitly target these processes—for example, graded exposure therapy designed to reduce pain-related fear and avoidance behavior, cognitive-behavioral interventions that train selective attention away from pain-relevant stimuli, and sensorimotor retraining paradigms that reinstate coordinated jaw motor control through progressive loading. Such multimodal approaches, which integrate attentional and sensorimotor rehabilitation targets, are increasingly prominent in orofacial pain management and may be particularly well-suited to address the specific cortical phenotype identified here.

### 4.1 Limitations

Several limitations warrant consideration. First, p-TMD status was established using a validated pain-screening questionnaire rather than an RDC/TMD Axis I clinical examination, which was not performed in the parent study (Wang et al., 2018, 2019, 2020). Diagnostic subtypes such as myofascial pain, disc displacement, and arthralgia could therefore not be characterized, and subtype heterogeneity may contribute unmodeled variance to the morphometric comparisons. Second, the modest sample size (N = 39) precludes definitive conclusions and limits power to detect smaller brain–behavior associations; no a priori power calculation was conducted for the morphometric analyses. Third, the p-TMD sample was predominantly male (13 of 19; 68%), inverting the female predominance typical of clinical and epidemiological TMD samples. This reflects the recruitment strategy of the parent study rather than the source population. Because sex did not differ between groups (χ^2^(1) = 0.74, p = .389), this is a matter of external validity rather than confounding of the between-group comparison; nonetheless, given documented sex differences in both pain processing (Bartley and Fillingim, 2013) and cortical morphometry (Ruigrok et al., 2014), the present findings may not generalize to the predominantly female clinical TMD population, and sex-stratified analyses were not feasible at this sample size. Fourth, variability in pain duration (range: 3–30 years) introduces heterogeneity that may obscure morphometric patterns specifically associated with pain chronification. Fifth, potential confounding variables—including comorbid chronic pain conditions, analgesic and psychotropic medication use, parafunctional behaviors such as bruxism, and laterality of jaw pain—were not systematically controlled. Sixth, the restriction of cortical analyses to a meta-analytic pain mask, while theoretically motivated, necessarily limits the potential for detecting differences outside this network. Seventh, structural images were acquired between 2009 and 2011, and although all processing was performed with a contemporary FreeSurfer release, the acquisition parameters predate current sequence conventions. Finally, the cross-sectional design precludes causal inference regarding whether the observed structural differences preceded, co-evolved with, or resulted from the chronic pain experience.

### 4.2 Conclusions

The present study provides preliminary evidence of localized cortical morphometric differences in the anterior and mid-anterior parts of the cingulate cortex in painful TMD, characterized by greater curvature and volume, respectively, in the absence of widespread cortical or subcortical structural alterations. The specificity of these findings to the aMCC is consistent with a model of regionally targeted neuroplasticity in chronic orofacial pain, although confirmation in prespecified, adequately powered studies is required.

## CRediT authorship contribution statement

**Soamy Montesino-Goicolea:** Writing – review & editing, Writing – original draft, Methodology, Investigation. **Pedro Antonio Valdes-Hernandez:** Writing – review & editing, Methodology, Investigation, Formal analysis. **Julio A. Peraza:** Writing – review & editing, Methodology, Investigation, Formal analysis. **Margarete Ribeiro Dasilva:** Writing – review & editing, Methodology, Investigation. **Stephen A. Coombes:** Writing – review & editing, Methodology, Investigation. **Yenisel Cruz-Almeida:** Writing – review & editing, Resources, Project administration, Methodology, Investigation, Conceptualization.

## Funding

This research received no grant from any funding agency in the public, commercial, or not-for-profit sectors. Participant compensation and magnetic resonance imaging costs for the parent study from which these data are drawn were met from internal 10.13039/100007698University of Florida funds. No funder had any role in study design, in the collection, analysis or interpretation of data, in the writing of the report, or in the decision to submit the article for publication.

## Declaration of competing interest

The authors declare the following financial interests/personal relationships which may be considered as potential competing interests: Yenisel Cruz-Almeida reports a relationship with United States Association for the Study of Pain Inc that includes: board membership. If there are other authors, they declare that they have no known competing financial interests or personal relationships that could have appeared to influence the work reported in this paper.

## Data Availability

The structural MRI data and clinical measures analyzed in this study were acquired under a parent protocol approved by the University of Florida Institutional Review Board (IRB #411-2007) that has since closed. The consent obtained at the time of data collection does not cover public deposition of individual-level data. De-identified data supporting the findings reported here are available from the corresponding author on reasonable request, subject to institutional approval and a data use agreement. The FreeSurfer processing pipeline and the meta-analytic mask used to constrain the cortical analyses are publicly available, as described in Methods 2.3 and 2.4.
